# Development of a Web-Based Experiential Learning Intervention for the Public to Reduce Cancer Stigma: Tutorial on the Application of Intervention Mapping

**DOI:** 10.2196/71166

**Published:** 2026-01-27

**Authors:** Miyako Tsuchiya, Keiichiro Adachi, Akiko Kimata, Kaori Kumagai

**Affiliations:** 1 Research Institute of Nursing, Musashino University, 3-3-3, Ariake, Koto-ku, Tokyo, 135-8181, Japan, 81 3-5530-7730; 2 Former Division of Health Care Delivery, Survivorship and Policy Research, Institute of Cancer Control, National Cancer Center, Tokyo, Japan; 3 Faculty of Health Sciences, Yamaguchi University Graduate School of Medicine, Ube, Japan; 4 School of Nursing, Mejiro University, Saitama, Japan; 5 School of Nursing and Social Services, Health Sciences University of Hokkaido, Ishikari, Japan

**Keywords:** cancer, digital intervention development, intervention mapping, stigma, survivorship

## Abstract

**Background:**

Stigma may negatively impact individuals throughout the continuum of cancer care and survivorship. Multitheory and multilevel intervention programs are necessary to reduce stigma but remain globally limited.

**Objective:**

This tutorial aims to illustrate the development of a web-based experiential learning intervention, “Friend Supporter,” designed for the public, which simulates scenarios to foster empathy and helping intentions. We applied the intervention mapping (IM) approach, which is rooted in the socioecological model, using the first four steps.

**Methods:**

In step 1, key issues faced by cancer survivors and influential factors were identified through empirical evidence and literature reviews on cancer stigmas and psychological theories. A multidisciplinary planning team assessed issue-related logic. In step 2, a logic model of change was created based on step 1 findings. In step 3, we designed program themes and a structure using systematic reviews and needs surveys among the public (n=1076) and cancer survivors (n=473), while applying theoretical change methods and practical strategies. Step 4 integrated prior findings. Inputs from an expert panel (n=5) and the public (n=13) using the think-aloud approach were used to refine the materials and functions, with educational resources for program providers also developed.

**Results:**

Step 1 revealed that public misconceptions and attitudes worsened the quality of life of cancer survivors. Step 2 identified 3 long-term outcomes: reduced public bias, improved responses to disclosure via acquired skills, and support aligned with survivors’ aspirations. The short-term primary outcome was helping intention. Personal factors (knowledge, skills, and self-efficacy) were also expected to improve helping intention as mediators. In step 3, the literature review showed that multicomponent online interventions effectively reduced stigma. The survey indicated the public most needed information on “how to interact with friends diagnosed with cancer” (317/1076, 29.5%), regardless of whether they had a friend diagnosed with cancer (*χ*^2^_1_=0.98; *P*=.32). Participants with no friends diagnosed with cancer were more likely to require information concerning “survival rates of all types of cancer” (*χ*^2^_1_=7.3; *P*=.007). Preferred delivery modes were booklets or leaflets (529/1076, 49.2%) and the internet (texts and figures: 460/1076, 42.8%). Cancer survivors wanted their friends to understand “the possibility of a cure as a result of early detection and treatment” (193/473, 40.8%). To produce program materials, we applied stigma and discrimination, protection motivation, social cognitive, and learning theories. The 5-module program included self-learning, role-plays, worksheets, and written feedback from clinical psychologists. Step 4 confirmed the feasibility of the program with minor refinement. We then developed a practical guide for program providers’ future implementation.

**Conclusions:**

IM is useful for systematically developing web-based multitheory and multilevel interventions. “Friend Supporter” offers a promising approach to enhance supportive behaviors and reduce cancer stigma. Quantitative evaluation is underway using the final 2 IM steps (implementation and evaluation) to determine real-world effectiveness.

## Introduction

### Background

Cancer stigma is a global public health concern, contributing to poor health and social inequality among survivors [1]. Stigma, which can be categorized as involving both public stigma and internalized stigma, is produced through complex societal processes [2]. The term “cancer” may automatically evoke fears of death (negative stereotypes or ignorance) or emotional discomfort (prejudice), which then may lead to discrimination toward cancer survivors. Survivors who are aware of this public stigma, in turn, may experience diminished self-esteem, intense self-blame, and shame [3], reflecting internalized stigma.

Public stigma undermines the cancer care continuum by reducing screening uptake, delaying diagnosis, and lowering treatment adherence, which can contribute to poor survival outcomes [1,4,5]. The literature further demonstrates that stigma negatively affects survivorship through impaired mental health [6-8], weakened social relationships [9], and reduced social participation [8,10]. Survivors of breast, cervical, prostate, and lung cancer are particularly vulnerable; the associations reported between these survivors and sexuality or risk-related behaviors (eg, smoking) may intensify both public and internalized stigma [3,7,9,11,12], further hinder timely access to care [4,8,13], and worsen quality of life (QoL) [3,14,15].

In Japan, however, policy interventions are not primarily aimed at reducing mortality through stigma mitigation. Instead, as articulated in the Fourth Basic Plan for Cancer Control [16], the national focus is on cancer survivorship through promoting coexistence with cancer by reducing stigma, enhancing public understanding, and fostering a supportive environment for survivors. This approach emphasizes citizen engagement and social inclusion to improve survivors’ QoL and to remove stigma-related barriers to social participation.

To reduce stigma, multitheory and multilevel interventions are recommended [13,17-20]. These approaches are grounded in the socioecological model, which emphasizes the dynamic interactions between intrapersonal factors and broader environmental contexts, such as interpersonal relationships, organizational structures, community settings, and policy frameworks [21]. Most illness-related stigma reduction programs focus on HIV/AIDS, leprosy, tuberculosis, mental illness or substance use disorders, and epilepsy [17-19,22], with limited numbers of cancer-specific intervention programs [1,23]. In Japan, 1 intervention dealing with romantic relationships among Japanese youngsters has been reported [24]. Most interventions rely on education and/or intergroup contact [17-19,21,24]. Sustaining intergroup contact poses challenges due to infectious risks (eg, COVID-19) and the burden on patients with cancer. Thus, interventions that minimize in-person contact while maintaining efficacy are needed.

Intervention content and delivery must also reflect relationships that survivors have (eg, with friends, colleagues, employers, teachers, caregivers, neighbors, and even those with no prior connection), survivors’ cancer types, and available provider resources (eg, human and infrastructure). However, developing multiple intervention versions for each context is impractical [25]. Instead, adapting core components may be a more feasible approach. We focused on friendships, which are vital to survivors’ well-being across the lifespan [26,27]. According to stigma research, engaging with hypothetical friends has been shown to help reduce psychological distance and foster empathetic understanding of an individual’s experience of stigma [28].

### Brief Description of the Intervention

We developed a 5-week web-based experiential learning program named “Friend Supporter” that was designed for the adult public with no history of cancer diagnosis (hereafter, the public). In this program, experiential learning is defined as a method that allows the program participants to learn the contents of the program and deepen their understanding of practical conduct through simulation. The program centers on a hypothetical scenario: “What if your friend were diagnosed with cancer?” This framing enables broad participation regardless of prior connection to cancer survivors as friends. The primary outcome of the program was helping intentions, assessed immediately after program completion. In addition, the program was designed to foster long-term outcomes, including supportive interactions with adult cancer survivors, increased support provision, and stigma reduction.

The intervention was conceptually designed for broad applicability across cancer types in adulthood, with a theoretical foundation that addressed common relational challenges and support needs identified in diverse contexts [29]. Through text-based role-play, program participants practice empathetic listening and supportive responses across diverse scenarios. These scenarios vary by cancer type, age, gender, occupation, disclosure context, and conversational content. These varied scenarios are intended to help participants adapt to different contexts. By applying core interpersonal skills learned through the program, they can respond more effectively across various situations. The program is grounded in the socioecological model and primarily focuses on intrapersonal and interpersonal levels, but with further potential effects at organizational and community levels.

### Objective

This paper aimed to illustrate the developmental processes of the 5-week web-based experiential learning program “Friend Supporter,” using the first 4 steps of the intervention mapping (IM) approach [30].

## Methods

### The IM Approach

#### Overview

IM, rooted in the socioecological model, is a systematic method for developing, intervening, and implementing health promotion programs by integrating theories, empirical evidence, and practical input [30]. IM emphasizes that interventions operate within dynamic systems, where their impact is influenced by interaction across environmental levels [30]. Thus, IM has been widely recommended for designing multilevel interventions [31]. This framework has been applied in diverse digital interventions; for example, pain self-management apps [32] and mobile-based self-compassion programs for patients with cancer [33], and web-based exercise interventions for patients with diabetes [34].

IM comprises 6 steps. Step 1 involves the identification of issues and factors, step 2 involves the establishment of program outcomes, step 3 involves program design, step 4 involves the production of materials, step 5 involves implementation planning, and step 6 involves implementation plan evaluation. Each step includes the completion of several tasks that form the basis for the subsequent step; however, the procedure is not always performed in a linear manner [30].

#### Step 1: Identification of Issues and Factors

In step 1, we identified issues in cancer survivors’ QoL and associated personal, behavioral, and environmental factors [30]. To do this, we conducted needs assessments focusing on breast cancer survivors [35,36]. This initial focus was chosen due to the availability of relevant Japan-based datasets and the stigma literature documenting relational challenges associated with breast cancer globally. We also investigated the public perceptions of cancer [37,38], existing literature reviews on cancer stigma [39], and relevant stigma and psychological theories [40-42]. This allowed us to provide a conceptual foundation for designing an intervention with broader applicability.

A multidisciplinary planning team was established that included a health psychologist, a clinical psychologist, a nurse, and an oncologist (another clinical psychologist and another nurse subsequently joined the planning team). Three members had the experience of information website development. All members discussed the logic model of the problem to reach a consensus.

#### Step 2: Establishment of the Program Outcomes

**Figure 1.**
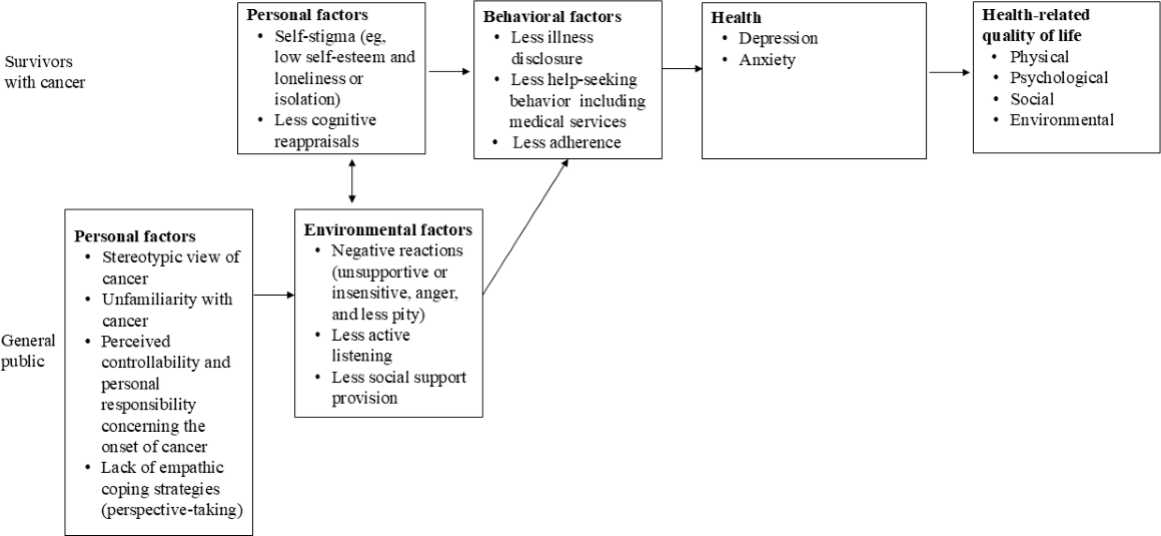
A logic model of the problem.

In step 2, we created a logic model of change that was intended to illustrate how the intervention program could modify or reduce the factors directly and indirectly associated with cancer survivors’ QoL, as identified in step 1; this model served as a theoretical framework rather than a definitive causal pathway [30]. As the logic model of the problem (Figure 1) shows, there were 2 possible populations for which the intervention program could be developed.

The multidisciplinary planning team discussed which populations needed to have interventions and what needed to be changed to improve the QoL of cancer survivors, in accordance with the national policy in Japan. Based on these discussions, the planning team decided to develop an intervention program designed for the public. This decision was grounded in the reality that many cancer survivors resume their usual lives and reintegrate into their communities after discharge. If a new intervention program could reduce stigma and discrimination among program participants, they might become advocates for cancer survivors. This, in turn, could empower cancer survivors and promote a society where living with cancer is more widely accepted.

A logic model of change was drafted to guide the intervention design. Behavioral and environmental impact, performance objectives (long-term outcomes), and change objectives (short-term outcomes) were established through a stepwise logic modeling process. Change objectives were mapped to personal determinants and organized in a matrix. These were theorized to support individual behavioral and interpersonal changes.

#### Step 3: Program Design

##### Overview

Step 3 involved generating themes, components, and sequences of components for the intervention program [30]. Theory-, evidence-, and practice-based change methods should be determined to optimize the effectiveness and adaptability of an intervention [30]. We reviewed existing systematic reviews and conducted 2 surveys (one among the public and another among cancer survivors) in Japan. This approach was adopted because customization based on user preferences facilitates smoother implementation [43]. To design the program, various theoretical and practical change methods were discussed among the planning team.

##### Existing Systematic Review of Literature

To identify existing effective interventions that could achieve the performance objectives established in step 2, the Cochrane Database of Systematic Reviews (CDSR) was searched. To gain insight into the intervention design and components, we reviewed interventions that were indicated in the findings of a meta-analysis as having significant effects.

##### Needs Survey Among the Public

To investigate the necessary content and preferable modes of intervention delivery, a cross-sectional online survey was conducted among the adult public (≥20 y) in Japan.

The detailed survey procedures have been previously reported in a study concerning a cancer-related scale development process among the public [44]. Potential adult Japanese participants were conveniently recruited from the panels of a research company. The company developed and tested the survey website for this study. Participants voluntarily accessed the closed survey and read the information sheets before participating. Each participant ticked the statement “I agree to participate in this study” before responding to the questionnaire. Question items were randomly presented to minimize order bias. The researchers created all the study materials except for the survey website and had no direct contact with the participants. The response rate was reported in a previously published article based on the same survey dataset [44]. As the recruitment and data collection were conducted by the research company, the investigators did not have access to individual-level invitation procedures and response tracking.

Data concerning demographics, willingness to disclose their cancer to surrounding people (families, friends, colleagues, and neighbors) if the participants were diagnosed with cancer, and whether they had friends with cancer were obtained. The participants were instructed to read a scenario describing a situation where a hypothetical friend talked about a cancer diagnosis, after which the participants were asked to respond to the following question: “Reflecting on your reactions to have better relationships with the friend with cancer, please tell us what you want to know?” There were 24 items with dichotomous responses. Additionally, a detail concerning preferred modes of delivery when the participants received the relevant information was requested.

The study variables were summarized using descriptive statistics (mean and SD or frequencies and percentages). Chi-square tests were conducted to examine differences in information needs and preferred modes of delivery between the participants whose friends had been diagnosed with cancer and those who had not in real life. Information needs were categorized into the relevant change objectives identified in step 2.

##### Needs Survey Among Cancer Survivors

To investigate the necessary content for the public, a cross-sectional online survey was conducted among adult cancer survivors in Japan. The detailed survey procedures have been previously reported in a study concerning cancer survivors’ psychological distress with cancer disclosure [45]. Potential adult Japanese participants were recruited from the panels of a research company, which were independent of those involved in the public survey. The recruitment process, consent process, survey design, and data collection methods were identical to those used in the public survey.

Demographic and cancer-related information was collected. The participants, including those who had been diagnosed with cancer 5 years previously and those who had already disclosed their cancer diagnosis to friends, were asked to respond to the following question: “To have better relationships, what information would you want your friends to know when you tell them of your cancer diagnosis?” There were 20 items with dichotomous responses.

The study variables were summarized using descriptive statistics (mean and SD or frequencies and percentages). Information needs were categorized into the relevant change objectives identified in step 2.

##### Setting Up the Theoretical and Practical Change Methods

Considering the findings from the CDSR search and the 2 surveys, the planning team discussed the appropriate mode of delivery and themes and sequences of the components of the intervention program. In parallel, theoretical change methods for each change objective were selected by referring to a taxonomy of behavior change methods in IM [46] where applicable. The planning team discussed various practical change methods to reach a consensus.

### Step 4: Program Materials

#### Overview

Step 4 involved drafting, pilot testing, refining, and finalizing the program material [30]. The planning team drafted and reviewed the initial content. While the evaluation framework was initially conceptualized during step 2 through the logic model of change, the relevant behavioral and environmental impact was further structured during step 4, as part of the intervention logic model. A multidisciplinary expert panel evaluated the conceptual and practical validity of the materials. The IT team deployed materials for pilot testing. The adult public participants engaged in think-aloud sessions and individual interviews. Based on these findings, we refined the materials, finalized the intervention logic model, and produced accompanying educational resources.

#### Internal Material Production

The planning team reviewed the theoretical and practical change methods identified in step 3 and proposed that the program consist of 5 modules corresponding to the themes and sequences established in step 3. Prior to the development of the module materials, the planning team established formatting and stylistic guidelines to ensure consistency and accessibility. These included: standardized font type and size across all modules; use of concise and short sentences written in polite Japanese; and active use of visual elements such as images, charts, and diagrams to support comprehension. These guidelines were applied throughout the material development process to ensure a coherent learning experience across modules.

The planning team was divided into subgroups according to their expertise (eg, medicine and nursing, and psychology). Each subgroup created specific component materials, using credible sources and empirical findings, where applicable. To facilitate the design and review process, materials were initially created in slide format (Microsoft PowerPoint), allowing for visual structuring and annotation. The fourth component of the program, which features role-playing scenarios, required a distinctive approach in relation to the other components. The program participants could choose from multiple cases and progress through them interactively. This component was visually prototyped to ensure clarity, usability, and flexibility. First-person narratives were used in role-play case stories, based on evidence from a systematic review showing their effectiveness in reducing mental health prejudice [47]. In the role-play case stories, a character (a cancer survivor) shares a personal experience via text on a screen (eg, “I’ve been diagnosed with cancer—take care of yourself too”). The program participants, acting as the character’s friend, are prompted to respond through text-based interactions, simulating a supportive conversation. Finally, the planning team independently reviewed all the initial materials, and their feedback was incorporated through iterative refinements.

#### Multidisciplinary Expert Inputs

A multidisciplinary expert panel (n=5), including a medical oncologist, a psychiatrist, an oncology nurse, and researchers in pedagogy and sociology, independently evaluated the intervention logic model and paper-based materials. Electronic versions with voice recordings in role-play components were also provided. Regarding the evaluation of the intervention logic model, 16 questions were developed and assessed using a 5-point Likert scale (completely agree/agree/neither agree nor disagree/disagree/completely disagree), referring to relevant logic model guides [48,49]. To evaluate the materials, 29 questions were similarly assessed using the same scale. Open-ended questions were also included to gather qualitative feedback. Responses to the Likert scale were summarized by frequency. To guide subsequent refinements, particular attention was given to comments associated with “disagree” and “completely disagree” responses.

#### Pilot Testing

After refinement reflecting the expert panel’s concerns, an IT team deployed “Friend Supporter” on the Internet. The planning team provided the IT team with detailed instructions regarding slide navigation and hyperlinking (in both PowerPoint presentation and PDF formats) to ensure that the materials could be deployed effectively. Revisions were conducted collaboratively through embedded comments within the slides, enabling iterative refinement. The program participants would be expected to complete one module per week using the internet, and modules they had already completed could not be reviewed.

To investigate the feasibility, usability, and acceptability of this web-based program, we conducted a pilot study using a think-aloud approach among the public in Japan (this study has already been published [50]). Participants were recruited using snowball sampling. Participant characteristics were summarized using frequency, and the interview transcripts were analyzed using content analysis. Based on the findings, the program was further refined, the intervention logic model was finalized, and educational materials for implementation were developed.

### Ethical Considerations

Ethical approval for the survey conducted among the public was obtained from the institutional research board of the National Cancer Center (2017‐378). Ethical approval for the survey among cancer survivors was obtained separately from the institutional research board of the National Cancer Center (2017‐134). For the 2 surveys, appropriate electronic informed consent was obtained from all participants. The surveys were anonymous. Participants in the public survey received a 2,000-yen gift voucher (approximately 17 USD at the time of study).Ethical approval for pilot testing was obtained from the institutional research board of the National Cancer Center (2021‐020). Electronic informed consent was obtained from all participants. Participants in the interviews received a 2,000-yen QUO card (a prepaid purchasing card), which was approximately 15 USD. No personally identifiable information, including initials, was included in the transcripts and the published results. All procedures were conducted in accordance with the ethical standards of the institutional research board and with the 1964 Helsinki declaration and its later amendments or comparable ethical standards.

## Results

### Step 1: Identification of Issues and Factors

The relevant literature shows that some members of the public have negative reactions toward cancer survivors due to beliefs about cancer (eg, “cancer equals death” and “incurable disease” [37]), lack of familiarity with survivors, and perceptions of the risk factors [37-39]. These interactions can adversely affect survivors’ self-concepts, cognitive reappraisals, social disclosure [35,36], help-seeking behaviors (eg, less medical use), treatment adherence [39], mental health status [39], and QoL [36]. Psychological theories such as stigma attribution theory [40,41] and empathetic coping theory [42] have been used to elaborate on the links between the general public’s attitudes and the impact of such attitudes on cancer survivors. The findings underscore the need to address cancer stigma across the cancer experience, including coexistence, early detection, and clinical care. Figure 1 illustrates the relationship among health problems, QoL, and associated factors. The multidisciplinary planning team agreed to this logic model of the problem.

### Step 2: Establishment of the Program Outcomes

The logic model of change was structured around three key components: performance objectives, change objectives, and personal determinants. This stepwise structure enabled a systematic evaluation of how targeted changes in personal determinants may lead to behavioral shifts, ultimately contributing to a more supportive environment for cancer survivors. While improved QoL of cancer survivors was the ultimate goal, this was not directly measured in this model.

To clarify the logic of change, the primary outcome was defined as strengthening the public’s intention to support hypothetical friends with cancer, representing the short-term learning effect of the program. This proximal change was considered a key indicator of immediate program success. To achieve the primary outcome, the intervention targeted intrapersonal mediators of change, such as increased knowledge about cancer and cancer survivors and greater self-efficacy, as well as interpersonal skills such as empathetic coping skills. Change objectives for each performance objective were presented together with personal determinants (knowledge, skills, self-efficacy, and attitudes) in a matrix; for example, regarding change objectives aimed at reducing stereotypes and prejudice about cancer and cancer survivors, personal determinants associated with knowledge focused on “increasing accurate knowledge of cancer and cancer survivors” (Multimedia Appendix 1). These short-term shifts were theorized to contribute to the long-term goals outlined in the three performance objectives and were expected to emerge over a span of 2 to 3 years.

### Step 3: Program Design

#### Existing Systematic Review of Literature

The CDSR search found that there were no systematic reviews about cancer stigma reduction interventions for the public. However, there was 1 article about mental illness stigma reduction interventions [47], which reported that mass media intervention, first-person narratives, and multiple-component interventions were effective in reducing prejudice about mental illness. Additionally, a meta-analysis revealed that 2 reviewed interventions [51,52] significantly decreased prejudice and increased knowledge of mental illness and of affected patients. These interventions included information about mental illnesses and relevant patients, addressing negative reactions toward such patients and providing practical guides to help these patients using patient-related stories. However, these interventions did not address communication issues shown in Multimedia Appendix 1 or the established outcome. The planning team decided not to adapt these existing programs in the cancer context.

#### Needs Survey Among the Public

A total of 1076 people participated in the survey. The descriptive statistics showed that the mean age was 47.2 (SD 11.4) years, 35.4% (381/1076) were women, and 17.5% (188/1076) had friends with cancer [44]. The participants reported that if they were diagnosed with cancer, they would “strongly agree/agree” to disclose their cancer diagnoses to their families (920/1076, 85.5%), friends (474/1076, 44.1%), colleagues (480/1076, 44.6%), and neighbors (104/1076, 9.7%).

After reading scenarios depicting hypothetical friends with cancer, the participants identified the following 5 most sought types of information: “how to interact with friends diagnosed with cancer” (317/1076, 29.5%), “types of cancer treatment” (288/1076, 26.8%), “side-effects of cancer treatment” (249/1076, 23.1%), “survivors’ desire for relationships with their friends” (212/1076, 19.7%), and “what cancer survivors do not want friends to say” (202/1076, 18.8%). These information needs were ranked and mapped to the relevant change objectives identified in step 2 (Multimedia Appendix 2). Chi-square tests revealed that the participants whose friends had not been diagnosed with cancer were more likely to require information concerning “survival rates of all types of cancer” (*χ*^2^_1_ =7.3; *P*=.007) compared with those whose friends had been diagnosed (Multimedia Appendix 3).

Preferable modes of delivery were booklets or leaflets (529/1076, 49.2%), the Internet (texts and figures: 460/1076, 42.8%; text only: 297/1076, 27.6%; text and video: 155/1076, 14.4%), apps (75/1076, 7%), DVDs (46/1076, 4%), and other (24/1076, 2%). There were no differences in these preferences between those participants whose friends had not been diagnosed with cancer and those whose friends had been diagnosed (Multimedia Appendix 4).

#### Needs Survey Among the Cancer Survivors

A total of 473 cancer survivors participated in the survey. The descriptive statistics showed that the ages ranged from 25 to 85 years, and that 49.7% (235/473) were men. The participants had primarily developed breast cancer (118/473, 24.9%), colorectal cancer (68/473, 14%), and prostate cancer (51/473, 11%), with most at the early stage (grades 0/I/II) of cancer (311/473, 65.8%) [45].

The 5 most important types of information that the cancer survivors wanted their friends to know included: “the possibility of a cure as a result of early detection and treatment” (193/473, 40.8%), “types of cancer treatment” (154/473, 32.6%), “survivors continuing their social life during/after cancer treatment” (130/473, 27.5%), “outpatient cancer treatment” (116/473, 24.5%), and “risk factors of cancer” (114/473, 24.1%). These information needs were categorized into the relevant change objectives identified in step 2 (Multimedia Appendix 5).

#### Setting Up the Theoretical and Practical Change Methods

According to the CDSR findings and the 2 surveys, the planning team decided to develop a multiple-component and web-based (texts and figures) intervention program. The contents of the program were to include information about interpersonal skills, cancer and its treatments, and cancer survivors’ social life, which would meet the needs of both the public and cancer survivors. Referring to the two reviewed interventions in the CDSR search [51,52], the structure and sequences would have 5 thematic components, in the following order: (1) cancer, treatment, and survivors; (2) emotional and cognitive reactions to hypothetical friends with cancer; (3) reasons for cancer survivors’ illness disclosure to friends and expected responses from them; (4) guiding principles and role-plays on how to listen to make survivors feel safe; and (5) survivors’ desire for relationships with and support from their friends.

Several behavioral change methods that matched the determinants and change objectives were selected. Regarding knowledge (increasing accurate knowledge of cancer and cancer survivors), details on stereotype-inconsistent information were derived from stigma and discrimination theory [53], and details on framing were derived from protection motivation theory [54,55]. Regarding skills (acquiring empathetic coping strategies to use when told about hypothetical friends’ cancer diagnosis), details on empathy training were derived from stigma and discrimination theory [56], details on modeling from social cognitive theory [57], and details on feedback from learning theories [58]. To facilitate understanding of how theoretical and practical change methods were selected and translated into the program components, a comprehensive table is presented in Multimedia Appendix 6. The planning team agreed to these practical change methods.

### Step 4: Program Materials

#### Internal Material Production and Refinements

After individually reviewing all the draft materials, the planning team observed notable variation in both the quantity and presentation styles across the 5 modules. Through discussions, the planning team decided to standardize the duration of each module to within 30 minutes and to clearly state the learning objectives at the beginning of each module to help program participants understand the intended goals.

#### Multidisciplinary Experts’ Inputs and Refinements

The multidisciplinary expert panel reported some disagreements concerning the validity of the intervention logic model and program materials. Regarding the logic model, disagreements were reported on “concrete descriptions of the activities” (n=1), “the impact does not deviate from the scope of the stated outcome of the activity” (n=1), “the approximate time is clearly stated in which program participants would complete each module of the activity” (n=3), and “the time until all the activities would be completed is clearly stated” (n=3). We added the estimated time to complete each module and the total duration required to complete the program.

Regarding the materials, disagreements were reported on the “appropriateness of content” (n=3), “appropriateness of sentence expression” (n=2), “cost effectiveness” (n=5), “usefulness for short-term outcomes” (n=2), “appropriateness of audio recording with case stories” (n=1), “appropriateness of the use of worksheets” (n=2), and “appropriateness of feedback for worksheets” (n=2).

Most disagreements of the expert panel concerned participant burden in relation to the program. The planning team prioritized the expert panel’s suggestions concerning addressing matters that might adversely affect the program participants’ learning motivation and understanding. Consequently, the volume of materials was reduced by deleting unnecessary quizzes and worksheets, and voice recordings of the case stories. Terminology (eg, the 5-y survival rate) was clarified by adding explanations, and layouts were refined by separating the contents into 2 slides. The planning team discussed the refined materials and reached a consensus.

#### Pilot Testing and Refinements

A total of 13 adults participated in the think-aloud session. The descriptive statistics showed that the ages ranged from 20 to 63 years. The program’s visualization, content, quantity, and written feedback on worksheets were well received among the program participants. However, some older participants expressed a preference for printed or PDF materials to review in advance [50].

**Figure 2.**
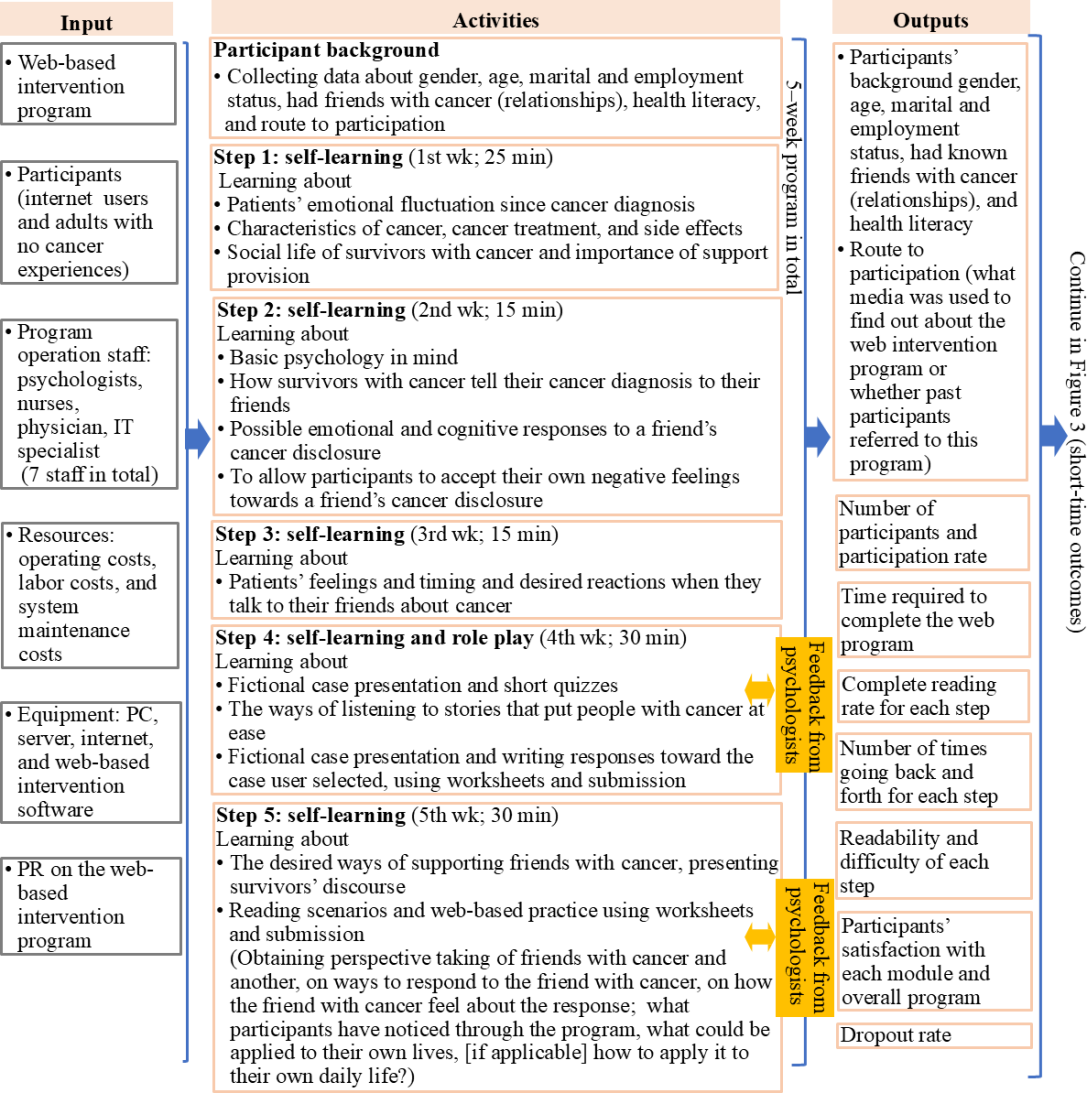
Intervention logic model: from input to output. PR: public relations.

The interview data (n=8) [50] showed that some participants preferred to revisit previously studied modules freely and requested access to external links to the case stories to explore a wider range of survivors’ experiences and situational contexts. Based on these findings, both printed and PDF materials were developed. The IT team added functions to allow program participants to freely browse complete modules. The planning team finalized the intervention logic model (the final version is shown in Figures2  3) and developed a practical guide for program providers to support future implementation.

**Figure 3.**
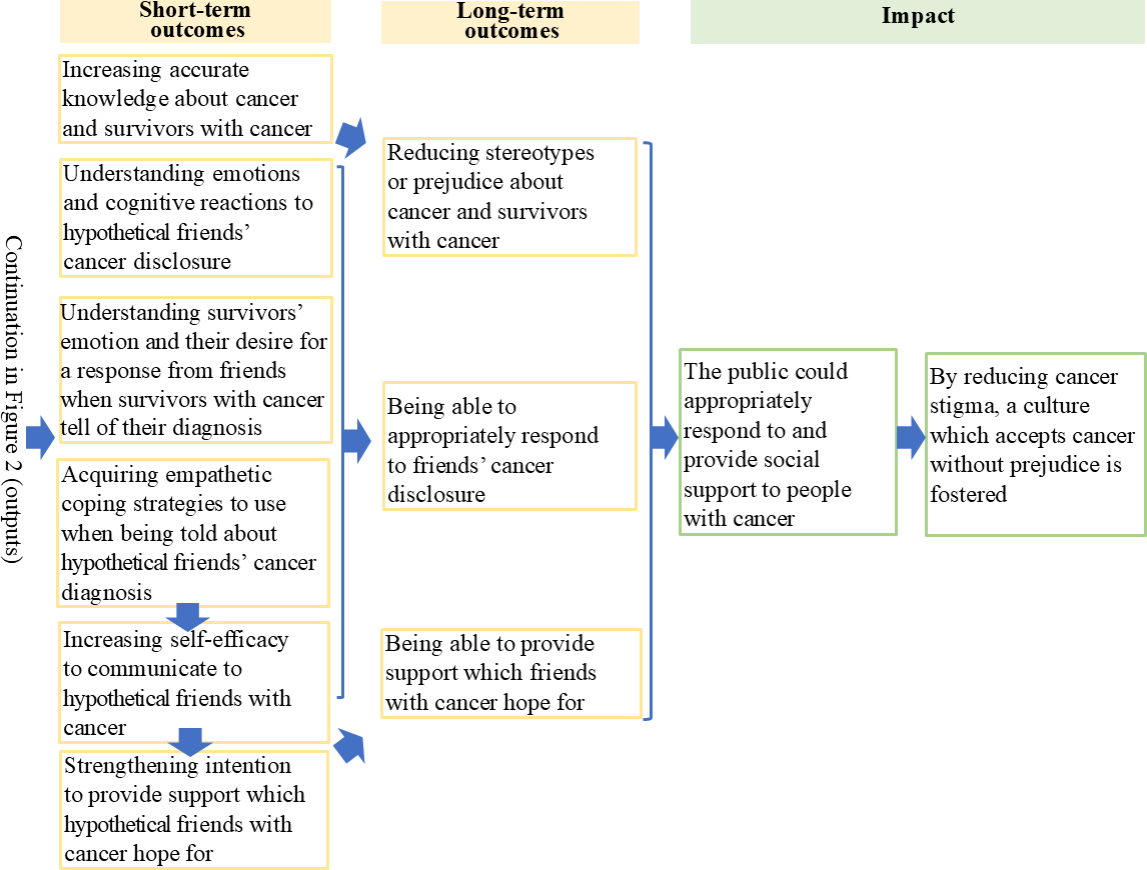
Intervention logic model: from outcomes to impact.

Furthermore, the interview data showed that the intervention program was found to reduce participants’ distancing attitudes and increase their intention to support hypothetical friends [50]. These promising results suggest the program’s potential to enhance helping behaviors toward cancer survivors (the snapshot of the “Friend Support” is shown in Multimedia Appendix 7).

## Discussion

### Principal Findings

This study is the first to apply the IM approach to develop a web-based experiential learning program aimed at reducing cancer stigma among the public.

We set out and described the first four steps of the IM approach [30]. While adapting existing evidence-based intervention programs can be more efficient than creating new ones, there remains a notable lack of programs aiming to reduce cancer stigma [1,23]. Therefore, it was considered worthwhile to undertake and report on a step-by-step developmental process that integrates empirical findings, behavioral change theories, and practical input. The incorporation of expert panel input and user feedback through a think-aloud approach significantly enhanced content refinement, as well as the relevance and feasibility of the final product.

The intervention logic model allowed for the development of the hypothesis that the primary goal of this proposed intervention program would be achieved, namely, to increase helping intentions toward hypothetical friends with cancer. We set up multiple mediators: increasing knowledge, empathetic skills, and self-efficacy. Individuals with heightened helping intentions are more likely to respond supportively when someone discloses a cancer diagnosis. Over time, such attitudinal changes may contribute to the three specified long-term outcomes: improved interpersonal responsiveness, increased support provision, and reduced stigma. If supportive behaviors become widespread across interpersonal, organizational, and community levels, the subsequent effects are more likely to foster a culture of inclusion, one that accepts cancer without prejudice and reduces discrimination against cancer survivors. While QoL was not explicitly defined as a direct outcome in the logic model, it serves as a foundational construct that underpins the broader objectives of the program. By fostering supportive environments and reducing stigma, the program is expected to indirectly contribute to enhancing the QoL of cancer survivors.

### Comparison With Prior Work

Prior studies have shown that public stigma contributes to internalized stigma among cancer survivors [6-14,59]. This dynamic aligns with the socio-ecological framework, which recognizes the interplay between societal attitudes and individual experiences. Whereas most digital interventions using IM have focused on intrapersonal factors to support individuals with cancer [32,33], our program uniquely targets the public, situated in the outer layer of the socio-ecological model, to promote broader cultural and relational change. Similar logic has been applied in intervention development targeting parental behavior, with the intention of indirectly influencing children’s physical activity levels by modifying relational dynamics [60].

Previous stigma reduction efforts have largely emphasized knowledge acquisition, including exposure to patient narratives [18-22,24]. However, social cognitive theory [61] suggests that knowledge alone may be insufficient to drive behavioral change; attitudes toward behavioral intentions are also essential components in predicting supportive actions toward individuals with cancer. Our experiential learning integrates empathy training together with cancer-related knowledge to foster emotional and cognitive empathy, and to enhance self-efficacy and helping intentions toward cancer survivors.

Unlike prior stigma studies that focused on specific cancer types [4,6,8,9,11-13,15,59], our program addresses stigma toward cancer survivors more broadly and is applicable to any kind of relationship. Centering on relational challenges and support needs common to diverse cancer contexts [29], the materials were intentionally designed for wide applicability. In addition to fostering transferable interpersonal skills, the program also equips participants with foundational knowledge about cancer, treatment processes, and the social lives of patients, namely survivorship. The program’s modular design allows for the inclusion of supplementary materials tailored to specific cancer types, enhancing its adaptability across diverse contexts.

### Strength and Limitations

This project demonstrates several key strengths in relation to the development of a web-based experiential learning intervention for cancer stigma reduction. First, the use of the IM framework enabled a systematic integration of theory, empirical evidence, and practical input. A multidisciplinary planning team guided the process through structured consensus-building, ensuring that diverse perspectives were reflected and that certain professional opinions were not overly influential. Second, the logic model helped identify and target essential mediators—knowledge, skills, and self-efficacy—within the program materials. The public survey data further informed the content and delivery preferences, aligning the intervention with the needs of both the public and cancer survivors. Appropriate reflection on the public’s needs, together with cancer survivors’ needs, may help motivate participation, implementation, and dissemination [43]. Third, the inclusion of communication-focused components and empathy training was undertaken in response to stigma-related challenges identified in prior research [56]. Various techniques, such as modeling, feedback, and first-person narratives [47], were incorporated to enhance self-efficacy and promote prosocial attitudes. The clinical psychologists’ written feedback on worksheets may provide further assurance and facilitate prosocial attitudes. Finally, expert reviews and end user feedback contributed to the credibility, feasibility, and relevance of the program, increasing the likelihood of achieving its performance objectives and supporting future implementation.

Regarding limitations, the investigation of the research on cancer stigma was limited to creating a logic model of the problem. However, recent findings suggest that public stigma may intersect with structural factors such as education and employment [10,62-64]. Future studies should address structural dimensions of stigma, which were not explicitly targeted in the current program. Further, we used the end user approach. However, while we did not invite cancer survivors into the expert panel, we compensated for this limitation by using the findings of the needs survey among cancer survivors. Finally, we did not establish a specific performance objective or change objectives for the environmental impact. In the future, appropriate change methods and practical applications should be identified and combined.

### Practical Implications

We plan to assess the effectiveness of this web-based intervention program, “Friend Support,” quantitatively, using randomized clinical trials among the public. If its effectiveness is confirmed, implementation studies could be performed in the future using steps 5 and 6 of the IM approach. To facilitate dissemination, intervention packages, including materials for users, a practical guide for program providers, and feedback training workshops for researchers and clinicians, have been prepared. A developed practical guide would be helpful for staff training, not only in terms of providing feedback on worksheets but also for understanding how to deliver the intervention program effectively.

For future implementation, collaboration with schools, companies, and community-based organizations, such as nongovernmental organizations or civil groups, could support localization and sustainable delivery. While the current program is framed around the role of a friend, extending this application to other social roles, such as workplace colleagues or teachers, may enhance its relevance. Core components of the program, particularly those related to interpersonal empathy and supportive communication, can be retained while adapting delivery methods to suit different contexts. For instance, incorporating reflective exercises that prompt program participants to consider how they might respond if a coworker or student were diagnosed with cancer could deepen empathy and foster supportive engagement in professional or institutional settings. These adaptations may also help address stigma in environments where social dynamics are shaped by hierarchical or role-based interactions.

The intervention has the potential to serve as a long-term resource for the public, promoting supportive attitudes and behaviors toward cancer survivors. Although the program was developed in response to Japan’s legal emphasis on stigma reduction in cancer survivorship, future research may explore whether stigma reduction can also encourage preventive behaviors, such as cancer screening and health care-seeking attitudes and behaviors [4,5,8,13].

### Conclusions

IM is a useful framework for integrating theory, evidence, and practical inputs to develop a web-based experiential learning intervention program designed for the public to reduce cancer stigma. A qualitative pilot test demonstrated the program’s feasibility, but further evaluation involving the public is required before implementation can be considered. We hope this tutorial paper will help researchers and educators systematically develop similar web-based experiential learning interventions, particularly those using simulation to address stigma and promote an inclusive and supportive community.

## Supplementary material

10.2196/71166 Multimedia Appendix 1 Matrix of the performance objectives and change objectives by personal determinants.

10.2196/71166 Multimedia Appendix 2 Information priorities in the public and corresponding change objectives (n=1076).

10.2196/71166 Multimedia Appendix 3 Information needs of the public in relation to having friends with cancer (n=1076).

10.2196/71166 Multimedia Appendix 4 Preferred mode of information delivery among the public in relation to having friends with cancer (n=1076).

10.2196/71166 Multimedia Appendix 5 Survivors’ knowledge priorities for friends and corresponding change objectives (n=473).

10.2196/71166 Multimedia Appendix 6 Theoretical methods and practical applications for change objectives and program components.

10.2196/71166 Multimedia Appendix 7 Snapshot of “Friends Supporter.”

## Abbreviations

CDSR: The Cochrane Database of Systematic Reviews
IM: intervention mapping
QoL: quality of life
