# Supplementary material for: Development of a Web-Based Experiential Learning Intervention for the Public to Reduce Cancer Stigma: Tutorial on the Application of Intervention Mapping
Source: JMIR Cancer. 2026 Jan 27;12:e71166. doi: 10.2196/71166 (PMC12840868; doi:10.2196/71166)
Supplement: Multimedia Appendix 1 [file cancer-v12-e71166-s001.pdf]

Multimedia Appendix 1 Matrix of the performance objectives and change objectives by personal determinants

Performance objectives	Change objectives by personal determinants			
	Knowledge	Skills	Self-efficacy	Attitudes
· Reducing stereotypes/prejudice about cancer and cancer survivors	· Increasing accurate knowledge of cancer and cancer survivors			
· Being able to appropriately respond to friends' cancer disclosure	· Understanding emotions and cognitive reactions to hypothetical friends' cancer disclosure<br>· Understanding cancer survivors' emotions and their desire for a response from friends when survivors tell of their diagnosis	· Acquiring empathetic coping strategies to use when being told about hypothetical friends' cancer diagnosis	· Increasing self-efficacy to communicate to hypothetical friends with cancer	
· Being able to provide support which friends with cancer hope for	· Understanding cancer survivors' emotions and their desire for a response from friends when survivors tell of their diagnosis<br>· Understanding survivors' desire for relationships with and support from friends	· Acquiring empathetic coping strategies to use when being told about hypothetical friends' cancer diagnosis	· Increasing self-efficacy to communicate to hypothetical friends with cancer	· Strengthening intention to provide support which hypothetical friends with cancer hope for
