# Supplementary material for: Development of a Web-Based Experiential Learning Intervention for the Public to Reduce Cancer Stigma: Tutorial on the Application of Intervention Mapping
Source: JMIR Cancer. 2026 Jan 27;12:e71166. doi: 10.2196/71166 (PMC12840868; doi:10.2196/71166)
Supplement: Multimedia Appendix 2 [file cancer-v12-e71166-s002.pdf]

Multimedia Appendix 2 Information priorities in the public and corresponding change objectives (n=1076)

Rank	Variables	Value<br>n (%)	Relevant change objectives
1	How to interact with friends diagnosed with cancer	317 (29.5)	S1, SE
2	Types of cancer treatment	288 (26.8)	K1
3	Side-effects of cancer treatment	249 (23.1)	K1
4	Survivors' desire for relationships with their friends	212 (19.7)	S1, SE1, A1
5	What survivors do not want their friends to say	202 (18.8)	S1, SE1, A1
6	Daily development progress of cancer treatment	190 (17.7)	K1
7	The possibility of cure as a result of early detection and treatment	188 (17.5)	K1
8	Incidence rates of cancer in Japan	184 (17.1)	K1
9	How to listen to make survivors feel safe	177 (16.4)	S1
10	Survivors' desire for support from their friends	170 (15.8)	S1, SE1, A1
11	Survival rates of all types of cancer	167 (15.5)	K1
12	What survivors want to hear from their friends	163 (15.1)	S1, SE1, A1
13	Distress till telling the illness to their friends	144 (13.4)	K1
14	Survivors continuing their social life during/after cancer treatment	138 (12.8)	K1
15	Outpatient cancer treatment	136 (12.6)	K1
16	Risk factors of cancer	135 (12.5)	K1
17	Survivors' positive experiences	122 (11.3)	K1
18	Fluctuation of survivors' feelings after cancer diagnosis	120 (11.2)	K1
19	Reasons for telling the illness to their friends	113 (10.5)	S1, SE1, A1
20	Difficulties and depression during and after treatment	93 (9)	K1
21	Psychological effects on survivors' family members	92 (9)	S1
22	How to deal with own emotions	83 (8)	K2

23	Economic effects on survivors' family members	82 (8)	-
24	Other	19 (2)	-

Survivors, survivors with cancer.

Change objectives: K1: Increasing accurate knowledge about cancer and survivors;

K2: Understanding emotions and cognitive reactions to hypothetical friends' cancer disclosure;

K3: Understanding survivors' emotions and their desire for a response from friends when survivors tell of their diagnosis;

K4: Understanding survivors' desire for relationships with and support from friends;

S1: Acquiring empathetic coping strategies to use when being told about hypothetical friends' cancer diagnosis;

SE1: Increasing self-efficacy to communicate to hypothetical friends with cancer;

A1: Strengthening intention to provide support which hypothetical friends with cancer hope for.
