# Supplementary material for: Development of a Web-Based Experiential Learning Intervention for the Public to Reduce Cancer Stigma: Tutorial on the Application of Intervention Mapping
Source: JMIR Cancer. 2026 Jan 27;12:e71166. doi: 10.2196/71166 (PMC12840868; doi:10.2196/71166)
Supplement: Multimedia Appendix 3 [file cancer-v12-e71166-s003.pdf]

Multimedia Appendix 3. Information needs of the public in relation to having friends with cancer (n=1076)

Variables	Having friends with cancer		Chi-square (df)	*P* value
	No (n, %)	Yes (n, %)		
Numbers of participants having friends with cancer	888 (82.5)	188 (17.5)	-	-
How to interact with friends diagnosed with cancer	256 (28.8)	61 (32)	.98 (1)	.32
Types of cancer treatment	225 (25.3)	63 (34)	5.3 (1)	.02
Side-effects of cancer treatment	201 (22.6)	48 (26)	.7 (1)	.39
Survivors' desire for relationships with their friends	169 (19.0)	43 (23)	1.4 (1)	.23
What survivors do not want their friends to say	163 (18.4)	39 (21)	.6 (1)	.45
Daily development progress of cancer treatment	148 (16.7)	42 (22)	3.4 (1)	.06
The possibility of cure as a result of early detection and treatment	153 (17.2)	35 (19)	.2 (1)	.65
Incidence rates of cancer in Japan	157 (17.7)	27 (14)	1.2 (1)	.27
How to listen to make survivors feel safe	144 (16.2)	33 (18)	.2 (1)	.65
Survivors' desire for support from their friends	140 (15.8)	30 (16)	.0 (1)	.95
Survival rates of all types of cancer	150 (16.9)	17 (9)	7.3 (1)	.007
What survivors want to hear from their friends	132 (14.9)	31(17)	.3 (1)	.57
Distress till telling the illness to their friends	119 (13.4)	25 (13)	.0 (1)	.97
Survivors continuing their social life during/after cancer treatment	115 (13.0)	23 (12)	.0 (1)	.79
Outpatient cancer treatment	104 (11.7)	32 (17)	3.96 (1)	.047
Risk factors of cancer	111 (12.5)	24 (13)	.0 (1)	.92
Survivors' positive experiences	104 (11.7)	18 (10)	.7 (1)	.40
Fluctuation of survivors' feelings after cancer diagnosis	97 (11)	23 (12)	.3 (1)	.60
Reasons for telling the illness to their friends	88 (10)	25 (13)	1.9 (1)	.17
Difficulties and depression during and after treatment	80 (9)	13 (7)	.9 (1)	.35
Psychological effects on survivors' family members	75 (8)	17 (9)	.1 (1)	.79

How to deal with own emotions	72 (8)	11 (6)	1.1 (1)	.29
Economic effects on survivors' family members	68 (8)	14 (7)	.0 (1)	.92
Other	15 (2)	4 (2)	.2 (1)	.68

Survivors, survivors with cancer.
