# Supplementary material for: Development of a Web-Based Experiential Learning Intervention for the Public to Reduce Cancer Stigma: Tutorial on the Application of Intervention Mapping
Source: JMIR Cancer. 2026 Jan 27;12:e71166. doi: 10.2196/71166 (PMC12840868; doi:10.2196/71166)
Supplement: Multimedia Appendix 4 [file cancer-v12-e71166-s004.pdf]

Multimedia Appendix 4 Preferred mode of information delivery among the public in relation to having friends with cancer (n=1076)

Variables	Having friends with cancer		Chi-square (df)	*P* value
	No (n, %)	Yes (n, %)		
Numbers of participants having friends with cancer	888 (82.5)	188 (17.5)	-	-
Booklets or leaflets	427 (48.1)	102 (54.3)	2.3 (1)	.12
Internet (texts and figures)	376 (42.3)	84 (45)	.3 (1)	.56
Internet (the text only)	239 (26.9)	58 (31)	1.2 (1)	.27
Internet (text and video)	125 (14.1)	30 (16)	.4 (1)	.51
Apps	67 (8)	8 (4)	2.6 (1)	.11
DVDs	38 (4)	8 (4)	.0 (1)	.99
