# Supplementary material for: Development of a Web-Based Experiential Learning Intervention for the Public to Reduce Cancer Stigma: Tutorial on the Application of Intervention Mapping
Source: JMIR Cancer. 2026 Jan 27;12:e71166. doi: 10.2196/71166 (PMC12840868; doi:10.2196/71166)
Supplement: Multimedia Appendix 5 [file cancer-v12-e71166-s005.pdf]

Multimedia Appendix 5 Survivors’ knowledge priorities for friends and corresponding change objectives (n=473)

Rank	Survey items	Value n (%)	Relevant change objectives
1	The possibility of cure as a result of early detection and treatment	193 (40.8)	K1
2	Types of cancer treatment	154 (32.6)	K1
3	Survivors continuing their social life during/after cancer treatment	130 (27.5)	K1
4	Outpatient cancer treatment	116 (24.5)	K1
5	Risk factors of cancer	114 (24.1)	K1
6	Survivors’ positive experiences	113 (23.9)	K1
7	Daily development progress of cancer treatment	107 (22.6)	K1
8	Incidence rates of cancer in Japan	97 (21)	K1
9	Fluctuation of survivors’ feelings after cancer diagnosis	91 (19)	K1
10	Survivors’ desire for relationships with their friends	84 (18)	S1, SE1, A1
11	Survival rates of all types of cancer	80 (17)	K1
12	Reasons for disclosing illness	74 (16)	S1, SE1, A1
13	How to listen to make survivors feel safe	67 (14)	S1
14	Economic impacts on survivors’ family members	62 (13)	-
15	Psychological effects on survivors’ family members	56 (12)	-
16	Survivors’ desire for support from their friends	45 (10)	S1, SE1, A1
17	Side-effects of cancer treatment	40 (9)	K1
18	What survivors do not want their friends to say	21 (4)	S1, SE1, A1
19	What survivors want to hear from their friends	10 (2)	S1, SE1, A1
20	Other	43 (9)	-

Survivors, survivors with cancer.

Change objectives: K1: Increasing accurate knowledge about cancer and survivors;

K2: Understanding emotions and cognitive reactions to hypothetical friends' cancer disclosure;

K3: Understanding survivors' emotions and their desire for a response from friends when survivors tell of their diagnosis;

K4: Understanding survivors' desire for relationships with and support from friends;

S1: Acquiring empathetic coping strategies to use when being told about hypothetical friends' cancer diagnosis;

SE1: Increasing self-efficacy to communicate to hypothetical friends with cancer;

A1: Strengthening intention to provide support which hypothetical friends with cancer hope for.
