# Supplementary material for: Development of a Web-Based Experiential Learning Intervention for the Public to Reduce Cancer Stigma: Tutorial on the Application of Intervention Mapping
Source: JMIR Cancer. 2026 Jan 27;12:e71166. doi: 10.2196/71166 (PMC12840868; doi:10.2196/71166)
Supplement: Multimedia Appendix 6 [file cancer-v12-e71166-s006.pdf]

Multimedia Appendix 6 Theoretical methods and practical applications for change objectives and program components

Program components	Change objectives by determinants	Theoretical Change methods	Practical applications
i) Cancer, treatment, and survivors	**Knowledge:** Increasing accurate knowledge about cancer and survivors	· Stereotype-inconsistent information<br>· Framing	Presentation of facts concerning cancer, risk factors, treatments, and survivors, using credible sources and positive framing words
ii) Emotional and cognitive reactions to hypothetical friends with cancer	**Knowledge:** Understanding emotions and cognitive reactions to hypothetical friends' cancer disclosure	Information	Presentation of emotional and cognitive reactions to hypothetical friends with cancer, using credible sources and research evidence, while emphasizing and not suppressing feelings and thoughts
iii) Reasons for survivors' illness disclosure to friends and expected responses from them	**Knowledge:** Understanding survivors' emotions and their desire for a response from friends when survivors tell of their diagnosis	Information	Presentation of survivors' emotions and their desire for a response from friends when survivors tell of their diagnosis, using research evidence
iv) Guiding principles and role-plays on how to listen to make survivors feel safe	**Skills:** Acquiring empathetic coping strategies to use when being told about hypothetical friends' cancer diagnosis	· Empathy training<br>· Modeling<br>· Feedback<br>· Experiential learning	Presentation of guided principles, case stories, and role-plays about good and not good responses or communication to hypothetical friends with cancer, using quizzes and elaborations.
iv) Guiding principles and role-plays on how to listen to make	**Self-efficacy:** Increasing self-efficacy to communicate to	· Empathy training<br>· Active learning<br>· Feedback<br>· Experiential learning	Presentation of case stories followed by worksheet-based reflection activities.

survivors feel safe	hypothetical friends with cancer		Submit structured worksheets and receive personalized feedback.
v) Survivors' desire for relationships with and support from their friends	**Knowledge:** Understanding survivors' desire for relationships with and support from friends	Information	Presentation of survivors' desire for relationships with and support from friends, using survivors' discourse obtained by empirical studies to show their individual differences
v) Survivors' desire for relationships with and support from their friends.	**Attitudes** :Strengthen-ing intention to provide support which hypothetical friends with cancer hope for	・Active learning<br>・Imagined contact<br>・Feedback<br>・Experiential learning	Engage in a reflective activity. Read vignettes and imagine the thoughts and feelings from both a person diagnosed with cancer and their friend. Submit structured worksheets and receive personalized feedback.

Survivors, survivors with cancer.
