# Supplementary material for: Development of a Web-Based Experiential Learning Intervention for the Public to Reduce Cancer Stigma: Tutorial on the Application of Intervention Mapping
Source: JMIR Cancer. 2026 Jan 27;12:e71166. doi: 10.2196/71166 (PMC12840868; doi:10.2196/71166)
Supplement: Multimedia Appendix 7 [file cancer-v12-e71166-s007.pdf]

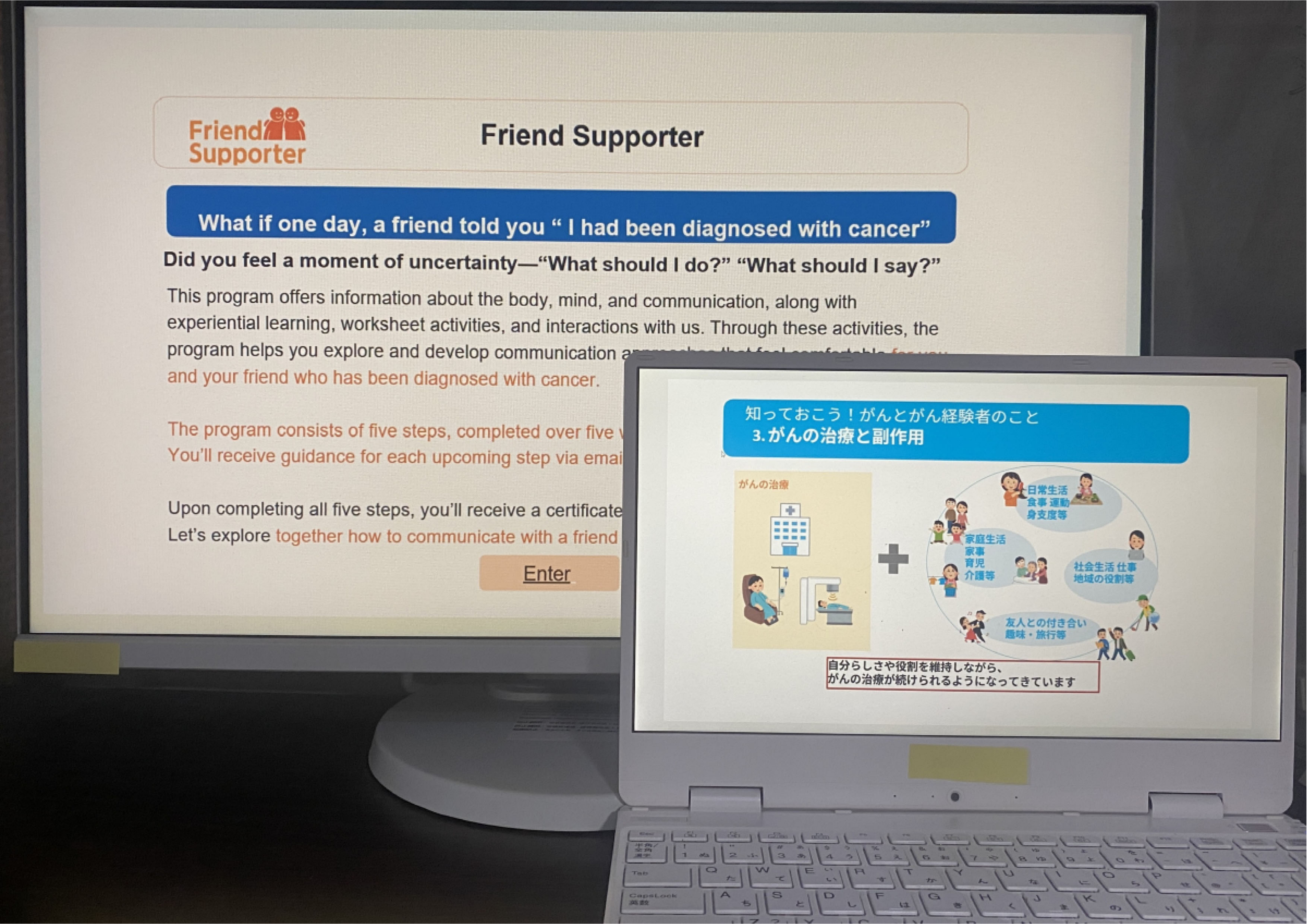
Friend Supporter
Friend Supporter
What if one day, a friend told you " I had been diagnosed with cancer"
Did you feel a moment of uncertainty—"What should I do?" "What should I say?"
This program offers information about the body, mind, and communication, along with experiential learning, worksheet activities, and interactions with us. Through these activities, the program helps you explore and develop communication
and your friend who has been diagnosed with cancer.
The program consists of five steps, completed over five
You'll receive guidance for each upcoming step via emai
Upon completing all five steps, you'll receive a certificate
Let's explore together how to communicate with a friend
Enter
知っておこう！がんとがん経験者のこと
3. がんの治療と副作用
がんの治療
日常生活
食事 運動
身支度等
家庭生活
家事
育児
介護等
社会生活 仕事
地域の役割等
友人との付き合い
趣味・旅行等
自分らしさや役割を維持しながら、
がんの治療が続けられるようになってきています
